# An Observational Study on the Effect of Yoga and Sudarshan Kriya in Type 2 Diabetes Mellitus Patients

**DOI:** 10.7759/cureus.27951

**Published:** 2022-08-12

**Authors:** Meenakshi R Verma, Deepakkumar G Langade, Rahul D Rao, Shreya Shivangi, Sonali Khedkar, Divya Kanchibhotla

**Affiliations:** 1 Conservative Dentistry and Endodontics, Bharati Vidyapeeth Dental College and Hospital, Navi Mumbai, IND; 2 Pharmacology, D. Y. Patil University School of Medicine, Navi Mumbai, IND; 3 Homeopathy, Lanco Hill's Tower, Hyderabad, IND; 4 Executive Director, Sri Sri Institute for Advanced Research, Mumbai, IND

**Keywords:** alternative therapy, living well, diabetes mellitus, sudarshan kriya, yoga

## Abstract

Introduction

One of the major health problems in many countries including India is type 2 diabetes. Yoga is being explored as an alternative therapy for the management of diabetes.

Methods

Among the total of 137 participants who successfully completed the ‘Living Well’ workshop by the Art of Living, 84 with type 2 diabetes were assessed for change in random blood sugar levels, systolic and diastolic blood pressure levels (SBP/DBP), and pulse rate over seven days. In the Living Well workshop, the participants performed a comprehensive Yogic Breathing Program that included yogic movements and postures (Asana), relaxation practice, three-stage Pranayama, Sudarshan Kriya Yoga (SKY), breathing techniques, and discussion of stress relieving principles over those seven days. The parameters were recorded at the time of enrollment and after the completion of seven days of the workshop.

Results

There was a greater (p<0.05) percent reduction in the mean blood sugar level in diabetics as compared to non-diabetic individuals. However, the reduction in SBP and DBP were similar (p>0.05) in diabetics and non-diabetics. The percent reduction in the pulse rate however was greater (p<0.05) in diabetics than non-diabetics.

Conclusion

Comprehensive yogic breathing practices comprising SKY may be beneficial in patients with type 2 diabetes mellitus.

## Introduction

Type 2 diabetes mellitus is a commonly seen lifestyle disorder. It is caused by insulin resistance with absolute or relative deficiency of insulin. This could result in chronic hyperglycemia and many cardiovascular complications. Unhealthy dietary patterns and sedentary habits are known to be a major risk factor for developing lifestyle disorders such as diabetes [1]. Psychological stress further adds to the increased risk and severity of the disease. Lack of any physical activity has been found to increase the risk of developing diabetes by three times and an increased risk of coronary heart disease by 2.4 times [2].

The science of *Yoga* and *Pranayama* has proved its usefulness in the treatment of certain diseases and for preservation of health in normal individuals. Studies have shown the useful role of yoga in the control of diabetes mellitus by reducing fasting and postprandial blood sugar level (BSL) significantly. The benefits of yoga therapy include long-term control of BSL, reducing requirement of medicines, and reduction in acute complications [3].

Short-term yoga-based lifestyle intervention has been shown to effectively reduce fasting plasma glucose levels in type 2 diabetes patients and pre-diabetes [4]. Other studies have shown a significant reduction in mean values of fasting as well as postprandial BSL in type 2 diabetes patients [5,6]. Completion and adherence to the practice of yoga has also shown to have a beneficial effect on diabetes in lowering HbA1c [7]. *Yoga Nidra* plus oral hypoglycemic agents have shown better control in fluctuating blood glucose and symptoms associated with diabetes compared to patients only on oral hypoglycemics [8]. Sudarshan Kriya Yoga (SKY) is a unique breathing technique developed by Art of Living (AOL), India, which balances the autonomic nervous system and improves the psychological and stress-related disorders [9]. Despite being practiced widely, there are limited data from India showing effect of yoga on random BSL in type 2 diabetes patients. The present study is observations made from participants who underwent a comprehensive yogic breathing program to assess the efficacy of SKY and yogic techniques for its blood glucose lowering effects in type 2 diabetes mellitus patients.

## Materials and methods

The current observational study was a single-arm study without randomization or blinding. Retrospective data of type 2 diabetes mellitus in adult participants over 18 years of age of both genders from the total of 137 participants who had participated and successfully completed the “Living Well” workshop of the AOL conducted over past four years were collected for analysis. All participants chosen had been screened for the presence of diabetes (based on their history) and were included in the study after obtaining written informed consent. The study protocol and study-related documents were reviewed and approved by the Institutional Ethics Committee (IEC) of Bharati Vidyapeeth Deemed University Dental College & Hospital, Navi Mumbai (Ref. No. BVDUDCH/IEC/133/2017; dt. 30 April 2017). The study was conducted in line with the principles of Good Clinical Practice (GCP) guidelines and the Declaration of Helsinki.

Patients under 18 years of age, pregnant and lactating women, and patients with type 1 diabetes mellitus or any condition, which could interfere with the administration of “yoga and sudarshan kriya” were excluded from the study. The patients enrolled in the study were assessed over seven days for the efficacy of SKY and yoga techniques of the Living Well course of AOL in the lowering effect on blood glucose as well as vital parameters (pulse rate and blood pressure) in patients with type 2 diabetes. At the time of enrolment, a detailed medical history of the participants was taken. Participants then underwent clinical examination, and vital parameters were recorded.

The living well workshop was for four hours every day for seven days. The comprehensive yogic breathing program included jogging and a drill walk followed by relaxation for two minutes. After relaxation, participants were asked to perform joint exercises (*sukshma vyayama*) of the neck, fingers, wrist, elbow, shoulder, hip, knees, and ankles for about 10 minutes [10]. After the exercises, all participants practiced *asanas* (back twist, forward and backward swing with “Ha” sound, side bending/*trikonasana*, *surya namaskar*, modified *padmasadhana*) for 30 minutes. The asanas were followed by three-stage *pranayama* (10/10/8) with *Ujjayi *or Victory breath, 3 sets of modified *bhastrika* or Bellows breath, *kapalbhati* (20-20-20 repetitions), straw *pranayama*, *nadishodhan pranayama* or balancing breath, and “Om” chanting for three times. The session ended with SKY [11]. After the *kriya*, participants were asked to relax for 15 minutes. The entire process was learnt under the guidance of an AOL-certified teacher. After seven days of the above, vital parameters, random BSL, and any adverse events were recorded. The duration of study for each participant was only seven days, and the entire study was conducted over a period of six months.

**Figure 1:**
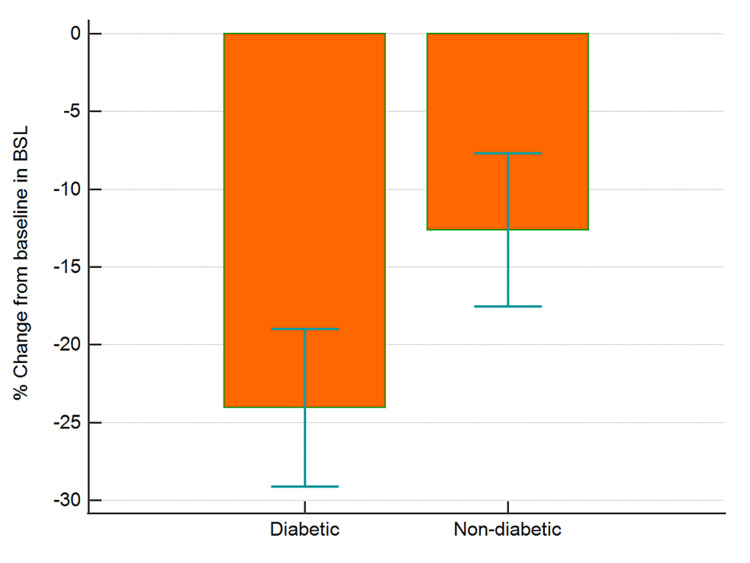
Percentage change from baseline in BSL in diabetic and non-diabetic participants Error bars are 95% confidence intervals for mean of % change BSL, blood sugar level

**Figure 2:**
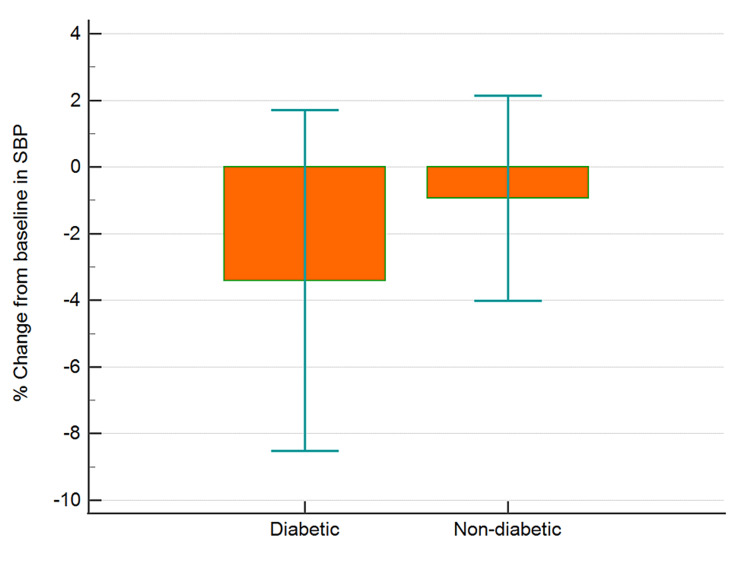
Percentage change from baseline in SBP in diabetic and non-diabetic participants Error bars are 95% confidence intervals for mean of % change SBP, systolic blood pressure

**Figure 3:**
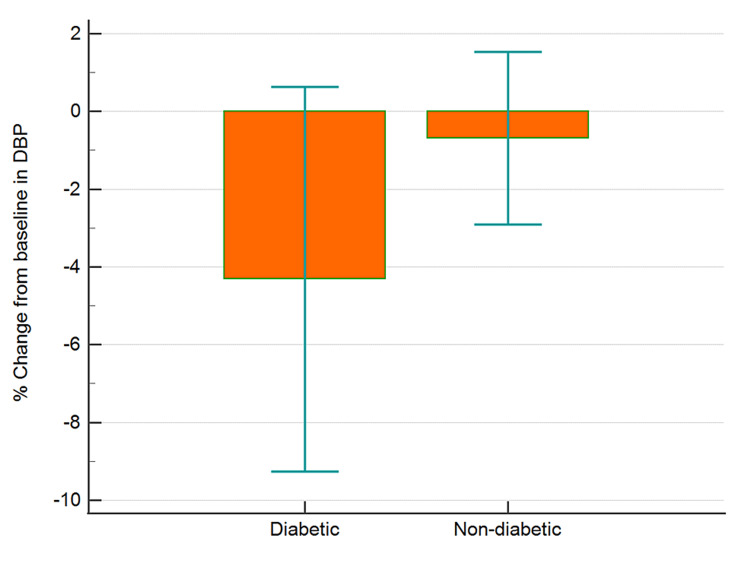
Percentage change from baseline in DBP in diabetic and non-diabetic participants Error bars are 95% confidence intervals for mean of % change DBP, diastolic blood pressure

**Figure 4:**
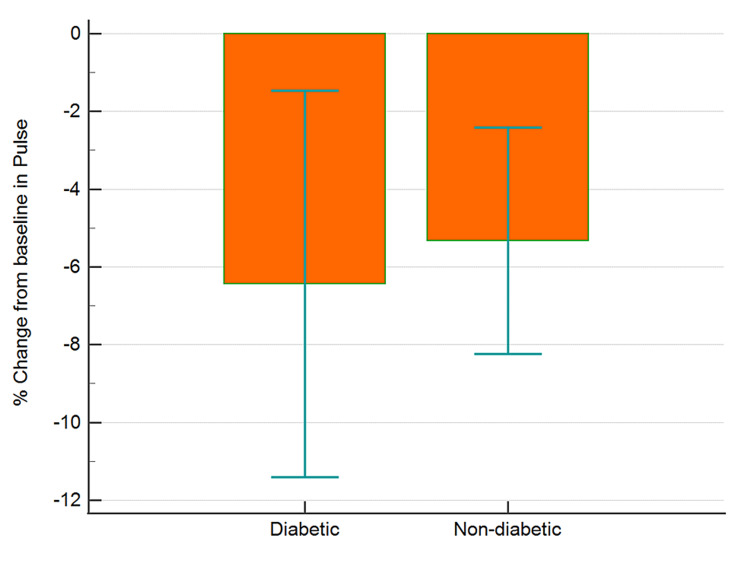
Percentage change from baseline in pulse rate in diabetic and non-diabetic participants Error bars are 95% confidence intervals for mean of % change

The data collected were cleaned and analyzed using MedCalc® Statistical Software version 20.015 (MedCalc Software Ltd, Ostend, Belgium; https://www.medcalc.org; 2021). Categorical data and discrete data are expressed as numbers with percentages. Continuous data are presented as means with SD. Baseline random blood glucose, pulse rate, and blood pressure readings were compared to post-therapy data using paired t-test. Between-group comparisons were analyzed using independent sample t-test. Percent changes from baseline values for blood sugar, blood pressure, and pulse rate were computed and analyzed for differences between the two groups (diabetic and non-diabetic) using independent sample t-test (Figures 1-4). An analysis of covariance was used for comparing the percent changes from baseline values for different parameters with hypertension, hypercholesterolemia, presence of back pain, and gender as covariates (Table 1).

**Table 1:** Percent change from baseline in BSL, SBP, DBP, and PR *ANCOVA (analysis of covariance) with hypertension, hypercholesterolemia, presence of back pain, and gender as covariates. **p-value of <0.05 is considered significant SBP, systolic blood pressure; DBP, diastolic blood pressure; PR, pulse rate; BSL, blood sugar level

	Diabetic (n=84)		Non-diabetic (n=53)		
	Mean	95% CI	Mean	95% CI	p-Value*
Blood sugar (mg%)	-22.85	-27.70 to -18.01	-12.59	-17.84 to -7.33	0.0057^**^
SBP (mm Hg)	-3.59	-7.95 to 0.77	-0.94	-5.62 to 3.74	0.4180
DBP (mm Hg)	-4.60	-8.66 to -0.54	-0.57	-4.92 to 3.78	0.1870
PR (per minute)	-6.51	-10.77 to -2.24	-4.95	-9.52 to -0.37	0.6260
Mean arterial pressure (mm Hg)	-4.24	-8.28 to -0.19	-0.84	-5.17 to 3.49	0.2630

## Results

A total of 137 participants were enrolled in the study from nine sessions, of whom 84 participants were diabetic and 53 were non-diabetic (Table 2). The study included 61.9% male and 38.1% female diabetic participants with a mean age of 50.35 years and a mean body weight of 72.7 kg. The baseline mean (SD) BSL was 164.38 (19.53) mg/dL, systolic blood pressure (SBP) was 133.43 (21.3) mmHg, diastolic blood pressure (DBP) was 82.98 (11.32) mmHg, and pulse rate (PR) was 86.11 (13.2) per minute.

**Table 2:** Demographics and baseline characteristics in the two groups *p-value of <0.05 is considered significant CVD, cardiovascular disease; SD, standard deviation

	Diabetic	Non-Diabetic	p-Value
No. of participants, N	84	53	
Male/female, N (%)	52/32 (61.90%/38.10%)	25/28 (47.16%/52.83%)	0.0916 (chi-square test)
Age (years), mean (SD)	50.35 (10.24)	48.69 (9.52)	0.3442 (independent sample t-test)
Body weight (kg), mean (SD)	72.70 (12.45)	76.46 (11.92)	0.0824 (independent sample t-test)
Hypertensive, N (%)	35 (41.66%)	18 (33.96%)	0.3692 (Fischer's test)
History of CVD, N (%)	14 (16.66%)	3 (5.66%)	0.0580* (Fischer's test)

Table 1 presents the mean (95% confidence intervals) values for percent change from baseline for BSL, SBP, DBP, and PR.

There was a greater (p<0.05) percent reduction in the mean BSL in diabetics as compared to non-diabetic individuals (Figure 1, Table 1). However, the reduction in SBP (Figure 2, Table 1), DBP (p>0.05), and PR (Figure 3, Table 1) were similar in diabetics and non-diabetics. The percent reduction in the PR however was greater (p<0.05) in diabetics than non-diabetics (Figure 4, Table 1).

## Discussion

Yoga has been extensively practiced since ancient times; however, as a therapy, it is still an emerging trend in the healthcare field. Diabetes mellitus is a significant problem affecting 69 million adult people. Many patients seek alternative therapy in the form of yoga and meditation [12]. The practice of yoga involves a complex intervention with various components that include a cleansing process known as *kriya*, controlled breathing known as *pranayama*, postures known as *asanas*, relaxation, and meditation, among others.

Many of these yoga practices have been found to be effective in the management of type 2 diabetes mellitus [13,14]. The result of the current study also proves the same. The reason attributed to this effect is a marked reduction in the BSLs, as is seen in the present study (p<0.05). Yoga practices are believed to increase insulin production and thus help in controlling diabetes. In the current study, the participants carried out jogging, *sukshma vyayams*, *asanas*, *surya namaskar*, relaxation, modified *padmasadhana*, a three-stage *pranayama*, *bhastrika*, *kapalbhati*, straw *pranayama*, *nadishodhan pranayama*, om chanting, and SKY.

*Asanas* focuse on synchronizing breathing and body movement. These involve stretching and twisting movements along with relaxation. Various postures stimulate secretion of insulin by pressurizing the pancreas. Keeping this in mind, the *asana* chosen was *Ardha Matsyendra Asana* (half fish pose), *Bhujang Asana* (cobra pose), *Dhanurasana* (bow pose), *Mandukasana* (frog pose), *Shalabhasana* (locust pose), *Yoga Mudra*, and *Shishuasana* (child pose), to name a few. It has been found that yoga postures have a positive effect on utilization of glucose and redistribution of fat in individuals having type 2 diabetes [15].

*Surya namaskar* or the sun salutation includes a series of postures performed in a defined sequence. It increases the cellular requirements for glucose and oxygen for which insulin production is stimulated [16]. In this study, the participants started with three sets of *surya namaskar*, and by day 7 they were doing seven sets, which also gave the participants benefit of a cardio workout.

*Pranayam* is a practice of controlled breathing. Slow *Ujjayi* breathing that was done by the participants in this study is said to enhance parasympathetic activity and increase indicators of vagal tone. It also decreases chemoreflex sensitivity, improves baroreflex response, and increases exercise and stress tolerance. *Ujjayi* breathing makes the practitioners feel calm [17]. The three-stage* pranayama* with *Ujjayi *breath is an advanced form of *pranayam* with specific ratios of inhalation, exhalation, and breath holds with specific arm positions. It controls the autonomic nervous system and regulates the heart rate. Three-stage *pranayam* helps to open all the three lobes of the lungs [18].

The breathing technique of *bhastrika* that was done is said to provide a mild sympathetic stimulation of excitation followed by emotional calming with mental activation and alertness. This increases the capacity of the sympathetic nervous system to respond to acute stresses without rapidly exhausting its reserves [11]. *Kapalbhati* is a technique of forceful exhalations and inhalations. This enhances the functional capacities of organs and improves the pancreatic β-cells efficiency. It is believed that this technique massages the internal organs resulting in an increased blood flow [19]. Om chanting has also been found to positively influence health. It is based on a mind-sound resonance technique that results in removal of negativity and an increase in energy [20].

SKY is an advanced rhythmic breathing technique of slow, medium, and fast normal breaths in cyclic succession. It has been found to significantly improve physical and psychosocial domains and the quality of life in patients with diabetes [21,22]. Previous studies suggest that SKY is useful for relieving depression [23,24] and improving antioxidant and immune defenses of the body [25].

According to the findings of the present study, it is seen that even short-term yoga practice has resulted in similar positive outcomes. The BSLs and PR were found to be reduced following yoga and SKY practice. The limitations of our study would be that it was a retrospective analysis, which only checked random sugar and not the fasting and postprandial glucose levels. Hba1c was also not measured in our study. A controlled comparative study between yoga and without yoga is needed for evaluating the true efficacy of yoga in the management of diabetes.

## Conclusions

Patients with diabetes commonly seek alternative therapy in the form of yoga for control of BSL. A seven-day course of yoga and SKY in “Living Well” program by AOL in patients with type 2 diabetes significantly lowered BSL and PR. SKY did not show any difference in blood pressure in diabetes patients. However, further large-scale multicenter studies for prolonged duration are needed to substantiate the beneficial effects of SKY in diabetic patients.
